# Sexually Dimorphic Effect of Environmental Enrichment and Weaning on Stress in Piglets

**DOI:** 10.3390/ani15081099

**Published:** 2025-04-10

**Authors:** Guadalupe Espejo-Beristain, Pedro Paredes-Ramos, Concepción Ahuja-Aguirre, Apolo Carrasco-García

**Affiliations:** Facultad de Medicina Veterinaria y Zootecnia, Universidad Veracruzana, Veracruz 91710, Mexico; gespejo@uv.mx (G.E.-B.); cahuja@uv.mx (C.A.-A.); acarrasco@uv.mx (A.C.-G.)

**Keywords:** animal welfare, behavior, pigs, sexual dimorphism, stress, weaning

## Abstract

Most intensive swine production systems use invasive practices that increase stress and compromise animal welfare. Apparently, the way in which animals respond to stress is sexually dimorphic. To cope with stress and improve the life of animals, environmental enrichment (EE) is used. The objective of the study was to evaluate how EE and weaning affect the behavior and cortisol levels of barrows and female piglets. Forty piglets were divided into two groups: (1) WEE: piglets that received EE from birth until 21 days of age and whose mothers received EE from week six of gestation until farrowing; (2) NEE: piglets for whom neither them nor their mothers received EE. Cortisol fecal metabolites and frequency, latency and duration of behaviors associated with stress were evaluated and compared before and after weaning. Before weaning, WEE barrows showed less inactive and ingestion behaviors and shorter duration of aggression (*p* < 0.05), while WEE females showed longer duration of aggression and higher frequency of ingestion behavior (*p* < 0.05). After weaning, WEE barrows and NEE females had higher cortisol levels, and NEE females showed longer duration of aggression (*p* < 0.05). In conclusion, piglets’ response to EE and the way they learnt to cope with stress were sexually dimorphic.

## Simple Summary

Worldwide, intensive swine production uses invasive practices that increase stress and reduce animal welfare. To reduce stress and improve the life of animals, environmental enrichment (EE) can be used. In this study, we evaluated if male and female piglets that received EE (toys) when they were with their mothers and their littermates responded differently to stress when they were weaned. The results showed that before weaning, the barrows that received EE were more active and less aggressive than the females that had EE and the barrows that did not have EE; also, the female piglets that received EE were more aggressive and ate more than the other piglets. After weaning, the barrows that received EE and the female piglets that did not receive EE were more stressed than the other piglets, and the female piglets that did not receive EE were more aggressive. Thus, it was concluded that the way the piglets respond to EE and stress, before and after weaning, depends on whether they are females or barrows; that is, this response is sexually dimorphic.

## 1. Introduction

Intensive swine production systems subject pigs to crowded conditions and sensory deprivation which lead to chronic stress and greatly affect their welfare. Although these practices guarantee large-scale pork production and cost reduction, they also severely limit the ability of the animals to express natural behaviors and to satisfy their basic needs [1,2,3].

Several studies have shown that the lack of stimulation and the intensity of the production systems can increase the manifestation of abnormal and stereotypic behaviors in the animals [4,5] and reduce the expression of species-specific behaviors, both of which increase stress [6]. In the short or medium term, stress can generate feeding alterations [7], cognitive impairment [8], and an increase in negative emotions [9], which diminish animal productivity [10] and immunity [11].

Some studies have demonstrated that the way animals, including humans, experience and cope with stress is sexually dimorphic, namely, it differs between females and males [12,13,14]. In mammals, the sexual differences can include anatomical and physiological characteristics, body condition, height, growth speed [15,16], canine teeth morphology [17,18] and behavior [19]. In pigs, before they reach 50 kg of weight, females have greater productive performance than barrows [20]. According to Power et al. [20], small-size barrows eat less and grow slower in the post-weaning period than females of their same age. Additionally, females grow faster during lactation, when they have access ad libitum to milk supplements [21]. Moreover, studies have shown that there are sexual differences in the pig brain, even in structures that are involved in the stress neuroendocrine response [22]; in fact, it is known that the hypothalamic–pituitary–adrenal (HPA) axis, which is the main participant in the stress response mechanism, is sexually dimorphic in mammals [23].

To improve the life conditions of animals under human care, some management programs have been implemented that include the use of environmental enrichment (EE). In general, EE consists of adding elements to the housing of the animals to increase and diversify the sources of stimuli [24,25]. Some studies have shown that animals that receive EE express a higher number of natural behaviors such as exploration, search, and play [9,25] and fewer abnormal behaviors [26], compared to animals that grow in an environment deprived of EE. In pigs, exposure to EE during gestation and the early postnatal period improves their performance in anxiety tests, as they display greater behavioral regulation compared to pigs that either do not receive enrichment during these critical developmental stages or receive it only later in life [27]. In addition, it has been observed that sows that receive EE during gestation show lower cortisol concentrations before farrowing and fewer behaviors associated with pain and anxiety during farrowing, compared to sows that did not receive EE [28].

The objective of the study was to evaluate if EE given during gestation and lactation influences the behavior and the stress response (measured through fecal cortisol metabolites) in barrows and female piglets, during lactation and after weaning.

## 2. Materials and Methods

### 2.1. Experimental Animals and Management

The study was conducted in a semi-intensive pig farm located in Veracruz, Mexico (Lat. 19°41′ N, Long. 96°56′ W), at an altitude of 165 m, with a mean annual temperature of 25.2 °C and annual precipitation of 2700 mm.

Forty piglets from different litters were included when they were 1 day of age. Pregnant sows and their piglets were chosen based on convenience and divided in two groups: (1) WEE (*n* = 20; 10 barrows and 10 females): piglets that received EE during lactation (days 1 to 21 of age) and whose mothers received EE since week 6 of gestation; and (2) NEE (*n* = 20; 10 barrows and 10 females): piglets that did not receive EE during lactation (days 1 to 21 of age) and whose mothers did not receive EE during gestation. We implemented EE during these specific periods based on a previous study [27]. At the farm, the lights were turned on daily at 6:00 a.m. and turned off at 8:00 p.m.

In accordance with standard farm management practices, piglet births were synchronized, and litters were mixed and redistributed on the day of birth to ensure that each sow nursed a similar number of piglets of comparable size. In our study, piglets were redistributed only within their experimental groups: piglets from WEE sows were fostered exclusively by other WEE sows, and likewise, piglets from NEE sows were fostered only by NEE sows. On the same day, piglets were ear-tagged for identification and housed with their mothers in individual maternity crates (2.5 m × 1.2 m). Each crate was equipped with a regular feeder and a drinker and had slatted elevated flooring at a height of 1 m. Inside each crate, at the front, there was a wooden box to keep the piglets warm through an electric heater.

The piglets suckled ad libitum starting at birth and additionally were offered creep feed (100 g daily per piglet) from days 7 through 21 of age. Following the farm’s routine management, males were castrated on day 6 of age. In accordance with common animal husbandry practices in Mexico, the procedure was performed without the use of anesthetics or analgesics.

On day 22, the piglets were weaned from their mothers and housed in groups of 20 individuals, together with piglets from different litters within their respective EE group (with or without), in 3.5 × 2 m pens equipped with two drinkers, one semi-automatic feeder, and slatted elevated flooring with a sloped cement base. Weaning took place between 8:00 a.m. and 12:00 p.m.

### 2.2. Environmental Enrichment

Sows from the WEE group received tactile stimulation in the back and sides using heavy-duty multi-purpose plastic gloves, a floor-cleaning plastic brush, and a paint roller covered with synthetic grass as described in Espejo et al. [28].

**Figure 1:**
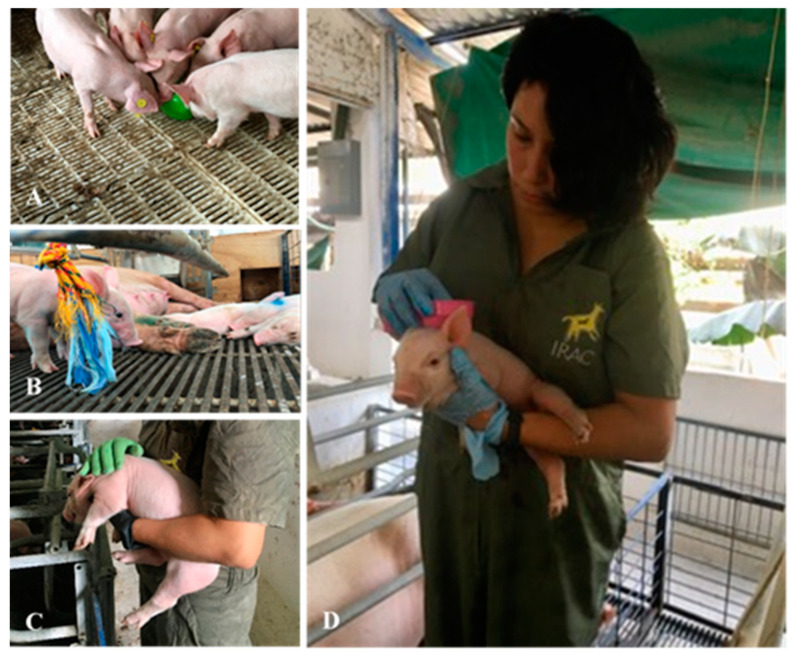
Some of the objects used as enrichment for the piglets: (**A**) plastic ball, (**B**) polypropylene/polyester rope, (**C**) massage with multipurpose glove, (**D**) massage with plastic brush.

The EE provided to the piglets of the WEE group consisted of (Figure 1) the following:

(1) Massages: one person picked up each piglet individually and provided them with tactile stimulation on their back and sides through one multi-purpose plastic glove and one domestic use plastic brush, both heavy-duty items.
(2) Balls: ten 10 cm-diameter soft plastic balls with no filling were provided and placed on the floor of the pen.
(3) Plastic hose: three 15 cm-pieces of ½ inch heavy-duty plastic hose tied together with a 40 cm cotton rope fixed to the pen walls.
(4) Rope: three 20 cm-pieces of 9 mm polypropylene/polyester rope tied together through a 40 cm cotton rope and fixed to the pen walls.
(5) Wood: 20 × 5 cm pine wood pieces, put together in a star shape with a 40 cm cotton rope.

The massages were given to the piglets during their first week of age for 30 s daily, in a head-to-tail movement pattern, with an approximate duration of 1 s per movement. The rest of the EE was offered daily for 30 min between 09:00 and 11:00 h during weeks 2 and 3 of age. The order of the EE was random, but it was always avoided to repeat the same EE in consecutive days.

The sows and their piglets from the NEE group did not receive EE.

### 2.3. Cortisol Fecal Metabolites

Three to five fecal samples were collected from each group of piglets on Days 20 and 25 of age, between 09:00 and 10:00 h. The samples were kept frozen at −20 °C until their analysis following the technique of Graham et al. [29]. Briefly, for the extraction of the cortisol fecal metabolites, the samples were thawed, dehydrated for approximately 2 h at 100 °C, and once dried they were pulverized using a mortar. From each sample, 0.5 g was taken and placed in a 10 mL plastic tube to which 5 mL of 80% MeOH was added to extract the steroid metabolites. The tubes were capped and placed in a shaker for 30 min. Then, the samples were centrifuged at 2500 rpm for 15 min and the supernatant containing the metabolites was recovered, placed in 2 mL plastic microtubes, and analyzed using commercial ELISA kits (DRG^®^ International, Inc., Springfield, NJ, USA) (Cortisol, DGR^®^, USA) for the determination of cortisol fecal metabolites. The assay showed cross reactivity with cortisol (100%), corticosterone (45%), progesterone (9%), deoxycortisol and dexamethasone (<2%), and oestrone, estriol, and testosterone (<0.1%). The range of the curve was 2.5–200 ng/mL. Assay sensitivity was 3.2 ng/mL. The intra-assay coefficient of variation was 1.08%. Cortisol values are expressed as ng/g of dry feces.

### 2.4. Behavior Analysis

The behavior of the piglets was video recorded using GoPro Hero 5^®^ cameras on days 21 and 22 of age for 30 min each between 11:00 and 12:00 h. The sampling technique used for registering the behavior was focal using an ethogram adapted from [30]. The latency, frequency, and duration of the behaviors described in Table 1 were evaluated.

### 2.5. Statistical Analysis

The levels of cortisol fecal metabolites, as well as the frequency, latency, and duration of the behaviors were evaluated through a generalized linear model (GLM-ANOVA). ANOVA was used to analyze the effects of the factors Group (with or without EE), Sex (male or female) and Weaning (before or after) on the dependent variable, with the variance among groups evaluated through the Levene test for the quality of the variances and the Tukey HSD test for the multiple comparison. The alpha value for all the comparisons was *p* < 0.05. The statistical packages used were Statistica v8 and Sigma Plot^®^ v11. All the data are presented as mean ± standard error.

## 3. Results

### 3.1. Fecal Cortisol Metabolites

**Figure 2:**
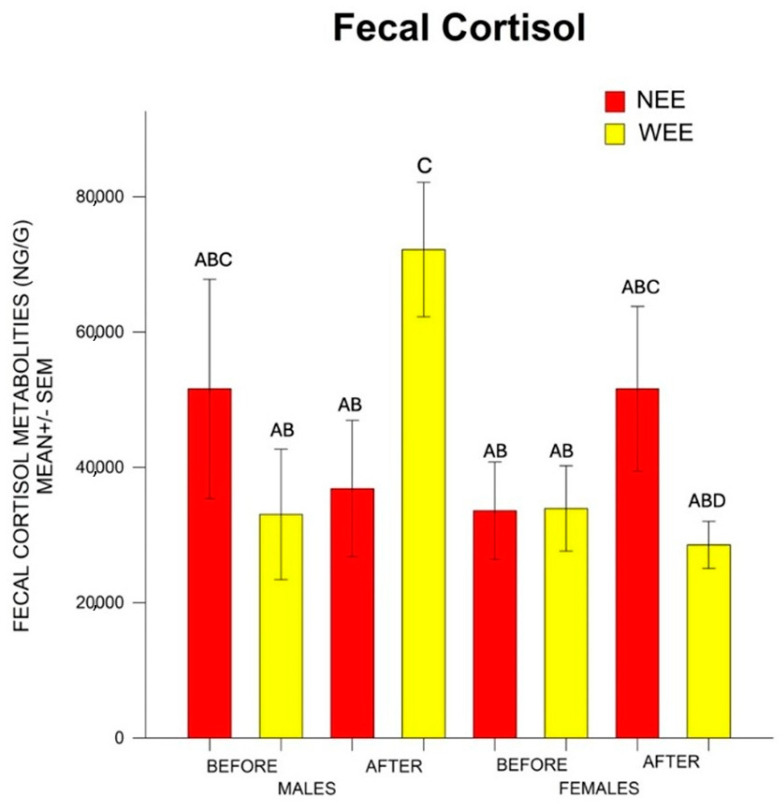
Mean ± SEM (standard error of the mean) of cortisol fecal metabolites before and after weaning in barrows and female piglets from the WEE and NEE groups. After weaning, the WEE barrows had higher cortisol than the rest of the piglets, while the NEE females had higher cortisol than the WEE females. Different letters (A, B, C, D) indicate statistically significant differences between groups (*p* < 0.05), as determined by Tukey’s post hoc test. Groups that do not share a letter are significantly different from each other.

There was no statistical difference in the factors Sex F = 2.32, *p* > 0.05; Group F = 0.038, *p* > 0.05; and Weaning F = 1.509, *p* > 0.05; or in the interactions Sex*Group F = 1.71, *p* > 0.05; Sex*Weaning F = 0.152, *p* > 0.05; and Group*Weaning F = 1.01, *p* > 0.32. Nonetheless, there was statistical difference in the interaction Sex*Group*Weaning F = 6.54 *p* < 0.05. The post hoc test indicated that after weaning, the barrows from the WEE group had higher levels of cortisol than the rest of the piglets. Regarding the females, after weaning, the NEE females had higher cortisol than the WEE females (Figure 2).

### 3.2. Behavior

The results of the interaction Sex*Group*Weaning of the behaviors analyzed are presented in Table 2. The post hoc test indicated that the frequency of inactivity and the duration of aggression was lower in the WEE barrows before weaning and in the NEE barrows after weaning. In addition, the NEE females showed a higher duration of aggression after weaning than the rest of females and barrows before and after weaning. The frequency of ingestion behavior was lower in WEE barrows and NEE females before weaning. The duration of ingesting behavior was the lowest in NEE barrows and the highest in NEE females after weaning.

## 4. Discussion

As expected, and in line with previous findings [27], EE during gestation and the early postnatal period was sufficient to influence the behavior and cortisol levels of piglets. However, unexpectedly, we observed sex-related differences in the impact of EE on piglet behavior during lactation and on cortisol levels after weaning. These results suggest that the effects of EE on the development of physiological and behavioral stress-coping abilities are sexually dimorphic in pigs.

In most animal species, there are sexual differences that appear and disappear during specific developmental periods and that are driven mainly by genetic and hormonal mechanisms [31,32], as the sex of an individual is determined by the presence of genes such as SRY, which is present in the male sex chromosome Y and absent in the female. A number of differences between males and females are reduced or increased as a consequence of learning and exposure to different hormones and environments [33].

The testosterone present in males since the embryonic stage and particularly at birth produces masculinization of the nervous system in males and increases the differences between males and females. Some studies have indicated that in the pig the differences can be observed since day 10 of gestation, causing the males to grow faster than the females until a few days before birth [34]. The postnatal testosterone present in male pigs increases in the first days of life and reaches a peak between the second and third week of life, diminishing progressively until three months of age [35]. This increase in testosterone enhances the differences between sexes and makes the males grow faster [36]. Unfortunately for males, these changes also seem to be related to a higher perinatal mortality and morbidity, caused mainly by crushing, thermoregulatory alterations, and a higher susceptibility to diseases in comparison with females [37].

Even though the males from the present study were castrated on day 6 of age following the routinary management of the farm where the study was conducted, it is acceptable to think that the action of the testosterone during gestation, birth, and the previous days to castration may have influenced the differences observed between barrows and females before and after weaning. In addition, neonatal castration itself may influence behavioral and cognitive outcomes, including responses to EE. For instance, one study [38] found that entire male pigs were more socially interactive, displayed more agonistic behaviors, belly-nosing, and showed greater interest in humans compared to castrates. Moreover, EE had differential effects depending on gonadal status: it reduced agonistic behavior in castrates and modified feeding and handling responses differently in castrates and entire males. These findings suggest that castration may alter how pigs perceive and benefit from enrichment, particularly in relation to social and exploratory behaviors. In our study, since all male piglets were castrated before the postnatal testosterone surge and no intact males were included, we cannot directly evaluate the role of gonadal status in shaping the observed responses to enrichment. This remains an important consideration for interpreting our results and for guiding future research.

The neonatal period is a critical stage of the socio-cognitive and emotional development of the animals; during this stage, the brain undergoes exponential and sudden growth that makes it more vulnerable to alterations, particularly if stress is present [39]. In consequence, the first experiences of life can determine the way in which animals will respond to internal and external stimuli during their lifetime [40]. A poor or inadequate environment can alter the social abilities of animals, as well as their capacity to cope with stress [41]. During the first weeks of life, pigs experience numerous challenges such as hierarchization with their littermates, fighting for their mother’s teats, weaning [42], and the adaptation to the change to solid diet [43], among others [44,45]. Also, the stress experienced by the sow during gestation can impact the development of the fetuses in the uterus, programming the physiology and the behavior of the offspring [46].

In the present study, the EE provided to the WEE piglets and their mothers should have given them a greater degree of welfare compared to the piglets and sows that did not receive EE. However, the behavioral analysis showed little differences between groups and no difference in the levels of cortisol before weaning. It was observed that the WEE barrows showed a lower number of inactive and ingestion behaviors, while the WEE females showed longer duration of aggression in comparison with the NEE females, as well as a higher frequency of the ingestion behavior. This suggests that EE during lactation particularly impacted the perception of welfare of the females, although not enough so as to modify the stress levels measured through cortisol fecal metabolites. The absence of differences in cortisol levels could have been because during lactation, the presence and interaction with the littermates and the mother was sufficiently enriching to the piglets of the NEE group, and this allowed them to regulate stress while they were not subjected to such a radical and challenging event such as weaning.

In natural conditions, pigs are weaned gradually from their mothers, sometimes until three months of age, which provides them with more social stimulation and allows them to acquire a progressive social and nutritional independence [47]. In the intensive production systems, weaning of the piglets occurs abruptly between 21 and 35 days of age [48]. Since that moment, pigs are grouped with individuals from other litters which, although the same age, differ in weight and size; this causes unbalanced fights in which the smallest or least experienced pigs get more injuries [49].

In the present study, the separation of the piglets from their mothers and the regrouping of the pigs after weaning had interesting sexually dimorphic effects. In the barrows, those which received EE showed higher cortisol levels than before weaning and the rest of piglets before and after weaning. This suggests that the aversive aspects of weaning were significantly more challenging and stressful for the WEE barrows than for the rest of the piglets. This phenomenon could be partially explained by the fact that during regrouping because of weaning, the males fight more than the females [50], although they are subdued by the females, which are more dominant [51]. In addition, the differences between WEE and NEE barrows could be because the first ones probably had fewer fights during lactation, reducing their opportunity to experience symbolic fights that normally prepare the animals to deal with future crises or crises from adulthood [52].

Finally, in the females, weaning only increased the cortisol levels in those which did not receive EE, and such increase coincided with a longer duration of the behavior of aggression manifested by these females in comparison with the rest of the piglets. This suggests that, unlike the barrows, the WEE females did develop more and better capabilities to cope with stress caused by the challenges from weaning and regrouping.

The results from the present study revealed sexual dimorphism in the piglets’ response to EE, which can have significant implications in the understanding of the animal welfare and management practices of pigs. Although EE has been associated with improvements in welfare, these findings indicate that its effects can vary considerably depending on the sex of the animal.

## 5. Conclusions

This study showed clear sexual dimorphism in the piglets’ response to EE and weaning. While the barrows experienced a higher level of stress after weaning, particularly those who received EE, the females with EE showed a better capacity to cope with stress, showing fewer alterations in the levels of cortisol and less aggressiveness. These findings highlight the importance of considering the sexual differences in the design of management strategies, as EE can have differentiated impacts on welfare depending on the sex of the individual.

## Figures and Tables

**Table 1:** Ethogram used for the behavior observations adapted from Luo et al. [30].

Behavior	Description
Laying inactive	Laying on their side or on their belly, with open or closed eyes, performing no other behavior.
Social exploration	Any physical and/or exploratory contact such as sniffing any part of the body of another piglet.
Pen exploration	Exploration and sniffing directed towards the pen walls, floor, feeder or drinker.
Social manipulation	Nibbling, sucking, chewing or biting at the ears, tail or another part of the body of another piglet.
Aggression	Thumping or pushing with the head or snout towards the body of another piglet.
Comfort behavior	Rubbing their body against objects or pen mates, scratching their own body with the leg or stretching the body quivering.
Ingestion	Eating and/or drinking water.
Locomotion	Continuous walk for at least 2 m.

**Table 2:** Mean ± SEM (standard error of the mean) and *p* value of the behaviors evaluated before and after weaning in barrows and female piglets with (WEE) and without (NEE) environmental enrichment. The latency (L) and duration (D) are expressed in seconds, while the frequency (F) is expressed as number of times. Values with different superscripts (a, b, c, d, bold font) differ significantly between groups (*p* < 0.05; Tukey’s post hoc test). Comparisons are made within rows.

Behaviour		Male Before Weaning NEE	Male Before Weaning WEE	Male After Weaning NEE	Male After Weaning WEE	Female Before Weaning NEE	Female Before Weaning WEE	Female After Weaning NEE	Female After Weaning WEE	*p*
**Laying inactive**	L	5.70 ±4.7	30.70 ± 5.8	310 ± 117.7	161.33 ± 62.9	42.50 ± 40.8	119.66 ± 49.1	313.90 ± 140.5	149.46 ± 53.2	0.73
	**F**	**4.70 ^a^± 0.73**	**2.80 ^b^ ± 0.52**	**3.20 ^b^ ± 0.4**	**4.06 ^a^ ± 0.32**	**4.70 ^a^ ± 0.6**	**4.16 ^a^ ± 0.4**	**4.80 ^a^ ± 0.4**	**4.20 ^a^ ± 0.3**	**0.046**
	D	1151.40 ± 140.1	1260.60 ± 238.2	722.20 ± 109.9	875.66 ± 73.8	1233.70 ± 122.5	995.76 ± 70.2	723.90 ± 150.5	954.40 ± 77.2	0.161
**Social exploration**	L	1180.20 ± 213.5	1105.33 ± 208.8	1697.80 ± 69.1	645.63 ± 118.3	1546.70 ± 168.8	1036.86 ± 147.6	1263.20 ± 231.9	574.43 ± 129.6	0.11
	F	0.90 ± 0.4	2.10 ± 0.39	0.20 ± 0.1	4.70 ± 1.11	0.20 ± 0.1	2.60 ± 0.5	0.80 ± 0.3	4.40 ± 0.6	0.42
	D	29.80 ± 12.06	131.86 ± 24.9	14.60 ± 12.3	303.70 ± 42.3	7.20 ± 6.07	147.90 ± 34.6	51.70 ± 28.5	251.00 ± 39.8	0.32
**Pen exploration**	L	1466.70 ± 120.7	1162.80 ± 219.7	300.90 ±100.4	965.43 ± 146.2	1257.00 ± 217.9	818.53 ± 147.5	851.20 ± 266.1	662.33 ± 149.4	0.18
	F	0.80 ± 0.32	0.80 ± 0.15	5.30 ± 0.6	1.90 ± 0.4	0.70 ± 0.3	2.03 ± 0.39	3.30 ± 0.9	2.76 ± 0.5	0.32
	D	60.50 ± 25.2	33.10 ± 6-2	432.60 ± 71.0	48.46 ± 12.2	54.70 ± 25.7	107.50 ± 30.4	251.10 ± 69.3	91.90 ± 18.1	0.11
**Social manipulation**	L	921.00 ± 227.0	1468.50 ± 277.5	797.80 ± 221.1	1596.90 ± 94.7	485.40 ± 157.6	1210.70 ± 142.5	1146.40 ± 227.7	1655.50 ± 80.03	0.30
	F	2.00 ± 0.6	0.40 ± 0.07	2.70 ± 0.5	0.36 ± 0.1	3.10 ± 0.7	1.26 ± 0.37	2.20 ± 0.7	0.26 ± 0.1	0.57
	D	169.50 ± 78.5	28.23 ± 5.3	261.20 ± 76.8	14.00 ± 8.06	165.50 ± 69.05	80.66 ± 30.4	154.80 ± 59.09	11.83 ± 7.1	0.70
**Aggression**	L	1800.00 ± 0	1579.70 ± 298.5	1297.30 ± 209.6	1355.50 ± 120.7	1293.30 ± 209.05	1087.00 ± 146.9	1108.80 ± 198.6	1103.60 ± 141.3	0.87
	F	0.55 ± 0	0.20 ± 0.03	0.80 ± 0.4	0.93 ± 0.2	0.50 ± 0.2	1.00 ± 0.2	1.20 ± 0.3	0.86 ± 0.2	0.36
	**D**	**1.10 ^b^ ± 0**	**0.70 ^b^ ± 0.13**	**2.20 ^a^ ± 1.2**	**2.50 ^a^ ± 1.3**	**0.80 ^b^ ± 0.3**	**3.80 ^a^ ± 1.2**	**7.30 ^c^ ± 2.8**	**2.50 ^a^ ± 0.6**	**0.05**
**Comfort behavior**	L	1702.00 ± 98	1800.00 ± 340.1	1800.00 ± 0	1733.30 ± 48.9	1800.00 ± 0	1697.20 ± 65.4	1800.00 ± 0	1617.40 ± 91.5	0.69
	F	0.10 ± 0.1	0.06 ± 0	0.01 ± 0	0.10 ± 0.07	0.06 ± 0	0.10 ± 0.05	0.06 ± 0	0.13 ± 0.06	0.41
	D	2.40 ± 2.4	0.01 ± 0	0.007 ± 0	5.80 ± 5.0	3.33 ± 0	1.50 ± 0.8	0.07 ± 0	7.70 ± 4.6	0.86
**Ingestion**	L	1075.20 ± 0.5	1066.20 ± 201.4	809.80 ± 224.6	617.80 ± 122.6	938.50 ± 207.9	778.03 ± 131.4	782.50 ± 213.01	454.96 ± 107.8	0.97
	**F**	**2.10 ^a^ ± 80.7**	**1.90 ^b^ ± 0.3**	**1.80 ^ab^ ± 0.5**	**3.40 ^a^ ± 0.4**	**1.60 ^b^ ± 0.4**	**2.90 ^a^ ± 0.4**	**3.10 ^a^ ± 0.6**	**3.46 ^a^ ± 0.4**	**0.05**
	**D**	**315.90 ^ab^ ± 0**	**221.16 ^a^ ± 41.7**	**180.50 ^d^ ± 56.7**	**395.40 ^b^ ± 47.9**	**273.10 ^a^ ± 85.4**	**289.83 ^a^ ± 44.6**	**415.00 ^c^ ± 104.7**	**371.46 ^b^ ± 42.1**	**0.043**
**Locomotion**	L	853.20 ± 220.4	858.76 ± 162.2	735.50 ± 86.5	416.06 ± 81.8	815.40 ± 219.2	672.30 ± 116.5	447.10 ± 183.2	475.86 ± 115.7	0.75
	F	2.70 ± 0.7	2.43 ± 0.45	4.70 ± 0.6	5.06 ± 0.4	2.50 ± 0.6	3.26 ± 0.37	6.00 ± 0.8	4.50 ± 0.5	0.10
	D	77.50 ± 32.0	124.00 ± 23.4	168.70 ± 28.7	159.23 ± 26.0	72.00 ± 32.6	166.80 ± 33.4	139.40 ± 32.4	107.40 ± 16.5	0.47

## Data Availability

Data are available upon justifiable request to the corresponding author.
